# Pedagogical questions promote causal learning in preschoolers

**DOI:** 10.1038/s41598-020-77883-5

**Published:** 2020-11-26

**Authors:** Emily N. Daubert, Yue Yu, Milagros Grados, Patrick Shafto, Elizabeth Bonawitz

**Affiliations:** 1 Rutgers University, Newark, USA; 2 National Institute of Education, Singapore, Singapore; 3 Present Address: University of Hawaiʻi at Mānoa, Honolulu, USA

**Keywords:** Human behaviour, Psychology

## Abstract

What maximizes instructional impact in early childhood? We propose a simple intervention employing “Pedagogical Questions”. We explore whether swapping some instructional language with questions in psychosomatic storybooks improves preschoolers’ memory, learning, and generalization. Seventy-two preschoolers were randomly assigned to one of three conditions and were read storybooks employing either *Direct Instruction*, *Pedagogical Questions*, or *Control* content. Posttest measures of psychosomatic understanding, judgments about the possibility of psychosomatic events, and memory for storybook details showed that children in the *Pedagogical Questions* condition demonstrated greater memory for relevant storybook details and improved psychosomatic understanding. Our results suggest that pedagogical questions are a relatively simple educational manipulation to improve memory, learning, and transfer of theory-rich content.

## Introduction

How can we maximize the impact of individual interactions to support learning? Science of learning principles emphasize cognitively engaging activities as paramount for children’s learning and memory^1–4^. However, there have been a limited number of proposals that improve learning outcomes and are simple enough to be implemented across a large range of scenarios. Here we investigate whether simply adding pedagogical questions—a specific type of child-directed question that may promote cognitive engagement—improves memory and learning.

We focus on a particular type of question, pedagogical questions, for our intervention. A pedagogical question is a question asked by a knowledgeable person, whose intention is to teach^5,6^. In contrast to questions like information seeking questions (when the questioner does not know the answer), pedagogical questions promote the discovery of target information, indicating that pedagogical questioning is perceived as “pedagogical” by children^7,8^. Like pedagogical questions, direct instruction also promotes the discovery of target concepts. However, direct instruction constrains exploration to the specific information provided by the teacher, whereas pedagogical questions both focus learners on critical content but also promote playful exploration and discovery^6–8^. We do not yet know how pedagogical questions influence learning, the retention of learned material, or the generalization of learned concepts.

To explore children’s learning of theory-rich content, we focused on the domain of psychosomatic reasoning, which is the understanding that psychological causes of physical outcomes exist (e.g., feeling frustrated can cause a headache^9^). Psychosomatic knowledge undergoes developmental change in the preschool years^9^, is relatively resistant to simple evidential interventions^10^, but is revisable given training that highlights causal mechanisms^11^. It is therefore an ideal domain to explore the implications of pedagogical questions for learning.

**Figure 1:**
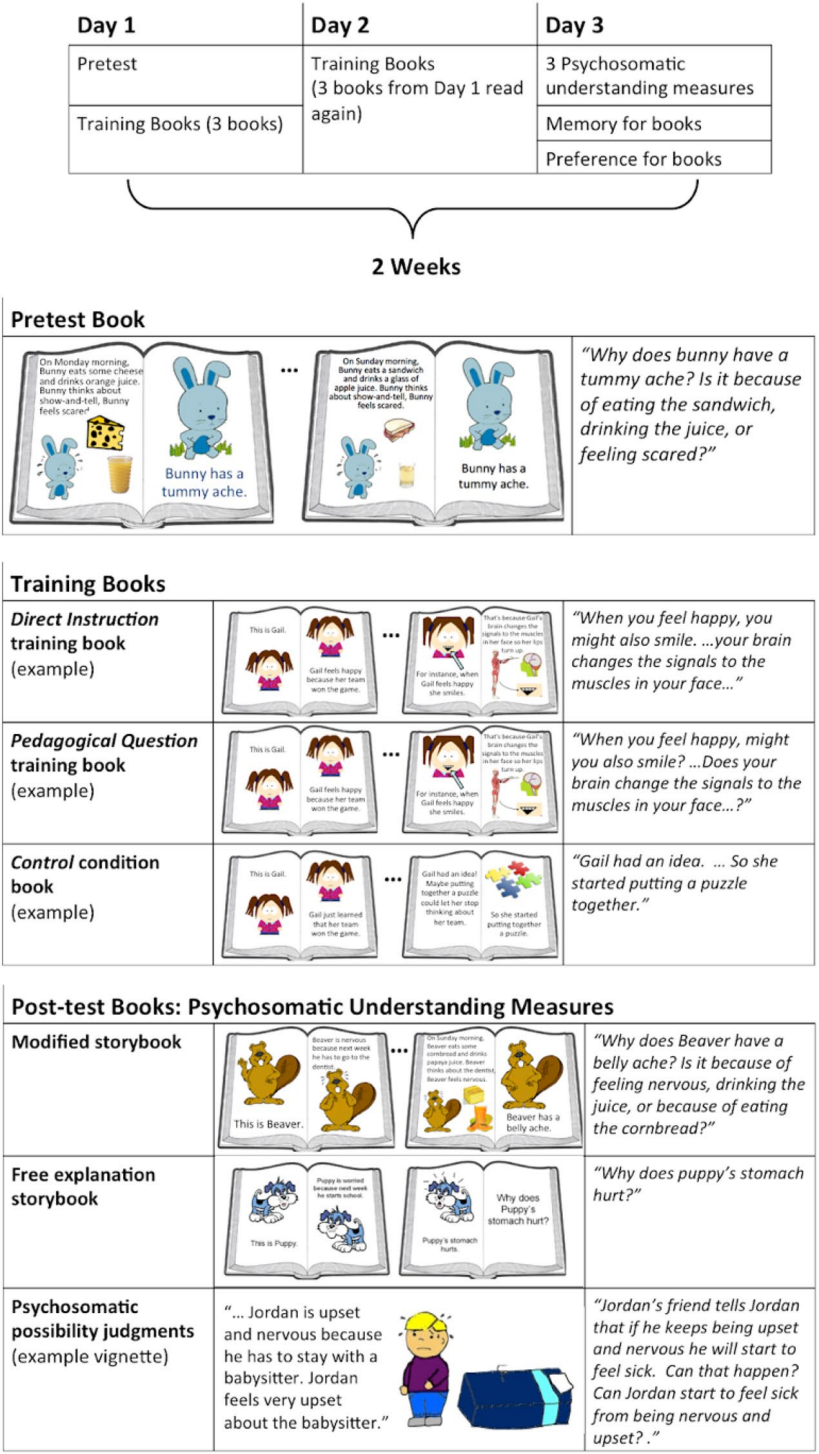
Experimental timeline and measures. Timeline, pretest, training/control books, and post-test examples. Storybook images adapted from “SP-Studio,” by J. Himmen (http://sp-studio.de). Copyright [2020] by Janina Himmen and SP-Studio. Adapted with permission.

We enrolled 72 preschoolers, ranging in age from 42- to 54-months-old (*M*_age_ = 49 months; 50% female), in a 2-week training study. Participants were randomly assigned to either the *Direct Instruction*, *Pedagogical Questions*, or *Control* condition. Over the course of 2 weeks, children completed all tasks one-on-one with an experimenter. All participants completed two training sessions and a posttest during three 15-min visits within a 2-week period. During pretest, participants read a screener storybook to ensure that they did not already endorse psychosomatic events. In this screener book, the protagonist (a bunny) repeatedly displayed a biological effect (having a tummy ache) after both biological events (eating and drinking certain things) and a psychological event (e.g., feeling worried; adapted from Ref.^10^; Fig. 1). The book was designed in such a way that statistically, the psychological event is the most likely cause of the biological effect. At the end of the book, children were asked to name the cause of bunny’s final tummy ache, and those who attributed the bunny’s tummy ache to a psychological cause (i.e., feeling worried) were considered to have “passed” the screening, and were subsequently dropped from the study and replaced.

Following the pretest, children were read three storybooks during a first visit, the same three storybooks during a second visit, and completed posttest measures during a third visit (Fig. 1). *Direct Instruction* and *Pedagogical Question* books highlighted psychosomatic causal mechanisms (as in Ref.^11^), where-as *Control books* did not focus on psychosomatic experiences. In all conditions, children were given informal prompts like, “Today we’re going to learn about Gail” to make it clear that children were engaging in an instructional interaction. The *Direct Instruction* books taught children possible causal mechanisms of psychosomatic events (adapted from the *Mechanism training* condition of Ref.^11^), and are written only in factual statement form.

The *Pedagogical Questions* books were identical to the *Direct Instruction* books, except that some of the statements were replaced with questions about the same information. For example, “When you feel happy, you might also smile.” in the *Direct Instruction* condition was changed to “When you feel happy, might you smile?” in the *Pedagogical Questions* condition. To ensure that the questions were interpreted as pedagogical, rather than information seeking, we included several factors, consistent with past studies of Pedagogical Questions (e.g. Ref.^5^). First, questions followed other declarative information about the causal mechanisms, demonstrating that the experimenter was knowledgeable. Second, the experimenter did not wait for the child’s response (which would be the appropriate action if a question was posed for the purposes of information seeking). Third, the inclusion of the question in the storybook itself is a demonstration of intent through purposeful design, providing an additional signal that the storybooks were intended to teach information. Taken together, these cues made it clear that the intention of the questions was pedagogical.

The *Control* storybooks followed the same protagonists and the same storylines as the *Direct Instruction* and *Pedagogical Questions* storybooks, except characters’ psychosomatic experiences were not discussed. Text in the control books contained emotion- and belief-related content as in the *Direct Instruction* and *Pedagogical Questions* books, but did not provide direct instruction or ask pedagogical questions about the causal content of psychosomatic events. Book length and the total number of words in each book were held constant across all conditions.

## Results

During posttest, children completed three measures of psychosomatic understanding: near generalization, free explanation, and far generalization. For the near generalization task, participants were read a new storybook with new characters that was structurally identical to the pretest book. Like the screener storybook, the modified storybook was designed in such a way that the psychological event is the statistically probable cause of the biological effect. Those who attributed the protagonist’s tummy ache to the psychological cause were considered to have demonstrated psychosomatic understanding.

**Figure 2:**
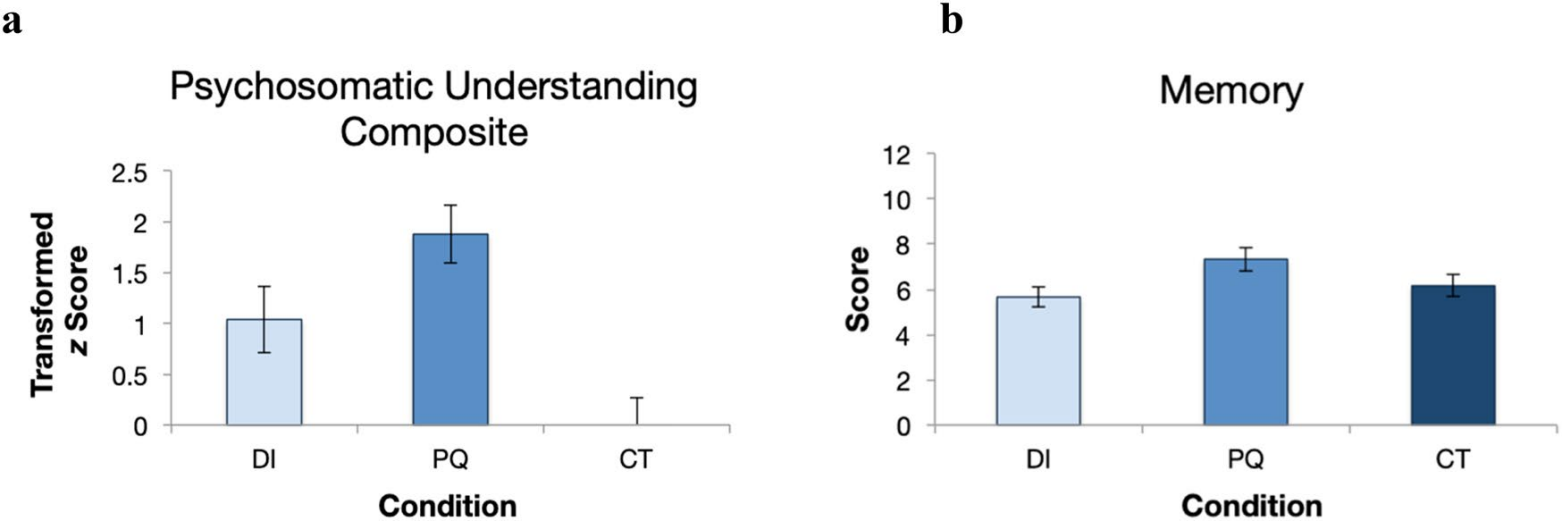
Psychosomatic understanding and memory measures by condition. (**a**) Composite of children’s psychosomatic understanding by condition. (**b**) Mean number of storybook details remembered by children in each condition.

A free explanation storybook (adapted from Ref.^11^) tested children’s ability to transfer knowledge to a novel task by asking children with an open-ended prompt for explanations for why a new character’s stomach hurt. Finally, children were given a far generalization task that required them to make judgments about the possibility of other psychological events causing physical effects. To assess improved psychosomatic understanding overall, children were given a composite score for near generalization, free explanation, and far generalization task (Fig. 2).

In addition to the measures of psychosomatic understanding, participants were asked questions to test their memory for details about the training books. These included three causally relevant questions (e.g. “Do you remember what happened to Gail in this story?”), as well as three questions on superficial, irrelevant content (e.g. “Do you remember her name?”).

Finally, we measured children’s overall preference for the storybooks. We provided children with a forced response task, in which we read the titles of each of three new books (PQ, DI, CT) with novel titles to the children, and then asked children which book they would prefer to read. Descriptive statistics are provided for all outcome measures in Table 1.

**Table 1:** Descriptive statistics for outcome measures.

Outcome measure	Direct instruction *M* (SD)	Pedagogical questions *M* (SD)	Control *M* (SD)
Psychosomatic understanding (transformed *z*-score)	1.04 (0.33)	1.88 (0.29)	0 (0)
Modified storybook	12.5% (6.9%)	37.5% (10.1%)	8.3% (5.8%)
Free explanation storybook	25% (9%)	33% (9.8%)	0% (0%)
Possibility judgments	2.88 (0.215)	2.38 (0.275)	2.00 (0.255)
**Memory for storybook details**	5.67 (0.51)	7.33 (0.488)	6.17 (0.433)
Relevant details	2.88 (1.624)	3.92 (1.692)	3.08 (1.558)
Irrelevant details	2.79 (1.250)	3.42 (1.349)	3.08 (0.881)
**Preference for storybooks (percent identified as first choice)**			
Direct instruction book	37.5%	41.7%	16.7%
Pedagogical question book	29.2%	20.8%	29.2%
Control book	33.3%	37.5%	54.2%

Means and standard deviations for children’s psychosomatic understanding, modified storybook and free explanation storybook performance, possibility judgments, and memory for storybook details by condition.

### Psychosomatic understanding

#### Composite score

We predicted that children in the *Pedagogical Questions* condition would score higher than children in the *Direct Instruction* and *Control* conditions, and that children in the *Direct Instruction* condition would score higher than children in the *Control* condition. As a preliminary step, we conducted two-tailed one-way analyses of variance (ANOVA) for all planned contrasts. For the composite psychosomatic comparison, there was a main effect of condition, *F*(2, 69) = 10.032, *p* = 0.00015. A planned contrast with weights of + 1 for Pedagogical Question, 0 for Direct Instruction, and − 1 for the Control condition was conducted. Children in the PQ condition scored higher than children in the DI and CT conditions, and children in the DI condition scored higher than children in the CT condition, *t*(69) = 4.47, *p* = 0.000015. These results suggest that the question-based storybook training, above and beyond direct instruction, helped children revise their entrenched prior beliefs about psychosomatic phenomena.

#### Modified storybook

We followed up with analyses for each of the psychosomatic measures individually. For the near generalization task with the structurally identical modified storybook, we predicted the same overall pattern such that more children would choose the psychological cause most in the *Pedagogical Question* condition and least in the *Control* condition. Children’s responses to the modified storybook were tested using an ANOVA, followed by a planned contrast with weights + 1 for PQ, 0 for *Direct Instruction* and − 1 for the *Control* condition. There was a main effect of condition for the near generalization comparison, *F*(2, 69) = 4.087, *p* = 0.021, indicating that children’s responses to the modified storybook significantly differed across conditions. Planned contrasts revealed that children in the PQ condition were more likely to identify the psychological cause (38%) than children in the DI (13%), and children in the DI condition were more likely to recognize a psychological cause than children in the CT (8%) conditions, *t*(36.553) = 2.509, *p* = 0.017. Furthermore, compared to children’s responses on the storybook screener (at floor due to the inclusion criteria), only children in the PQ condition were more likely to appeal to psychosomatic causes (37.5%, Fisher’s Exact, *p* = 0.002) during the modified storybook at posttest. Children in the DI (12.5%, *p* = 0.234) and CT (8.3%, *p* = 0.489) conditions did not significantly improve their performance.

#### Free explanation storybook

In the free explanation storybook, which tested children’s ability to transfer their knowledge to a novel task, we predicted that children would mention a psychological cause most in the *Pedagogical Questions* condition and least in the Control condition. Children’s responses to the free explanation storybook also significantly differed across conditions, *F*(2, 69) = 5.068, *p* = 0.009. Planned contrast with weights + 1 for *Pedagogical Questions*, 0 for DI, and − 1 for the *Control* condition revealed that children in the *Pedagogical Questions* condition were more likely to identify the psychological cause (33%) than children in the *Direct Instruction* (25%), and children in the *Direct Instruction* condition were more likely to recognize a psychological cause than children in the *Control* (0%) conditions, *t*(23.000) = 3.391, *p* = 0.003.

#### Psychosomatic possibility

Children’s judgments about the possibility of psychological events causing physical effects were also measured. Consistent with past work that did not find an effect of relatively short training on far generalizations, children’s judgments about the possibility of psychosomatic events in our task was not significantly different between conditions, *F*(2, 69) = 0.752, *p* = 0.475; children failed to endorse broader psychosomatic events following the brief training.

### Memory for storybook details

In addition to psychosomatic understanding, we also measured children’s memory for storybook details. We hypothesized that pedagogical questions would lead to better recall of details in the training books because they involved engaging questions during the books, whereas both the Direct Instruction and Control books were didactically read to the participants. Children’s memory for storybook details was first tested using a two-tailed one-way ANOVA on total memory score; there was a main effect of condition, *F*(2, 69) = 3.205, *p* = 0.047. A planned contrast with weight + 2 for Pedagogical Question and − 1 for the other two conditions revealed that children in the *Pedagogical Questions* condition remembered significantly more details about the storybooks than children in the *Direct Instruction* and *Control* conditions, *t*(69) = 2.421, *p* = 0.018.

#### Relevant versus irrelevant storybook details

Focusing only on relevant story details (i.e. what happened to characters), children’s memory was marginally significant across conditions, *F*(2, 69) = 2.760, *p* = 0.070. Planned contrasts revealed that children in the *Pedagogical Question* condition remembered significantly more details about the storybooks than children in the *Direct Instruction* and *Control* conditions, *t*(69) = 2.307, *p* = 0.024. In contrast, there was no main effect of condition on children’s memory for irrelevant story details (i.e. character’s names), *F*(2, 69) = 1.694, *p* = 0.191. Planned contrasts revealed that there were no significant group effects for irrelevant story details, *t*(69) = 1.628, *p* = 0.108 indicating that the group differences in children’s memory for storybook details were driven by relevant story details only.

### Children’s storybook preferences

Finally, we analyzed whether children’s learning was simply a result of their preferences for certain storybooks over others. Children expressed no systematic preference for any of the storybooks, *Χ*^*2*^(4) = 4.52, *p* = 0.359.

## Discussion

It is critical to develop effective and simple interventions that facilitate children’s understanding of complex real-world domains. Our results suggest that a particular intervention, asking pedagogical questions, offers a simple method by which we might engage young learners. A relatively minor modification to a storybook led to significant improvement in preschool-aged children’s learning, generalization, and memory of theory-rich content. Specifically, children who received pedagogical questions during training were better able to identify the prior belief-violating, yet statistically probable psychological cause. Further, despite having no preexisting preference for storybooks containing pedagogical questions, these participants were best able to bring their understanding of psychosomatic events to bear on the free explanation task. Additionally, children who heard pedagogical questions remembered more details about the training storybooks (e.g. characters’ names and specific events) than children in the *Direct Instruction* and *Control* groups.

In this study, the pedagogical question condition, by design, was actually a hybrid condition that included both direction instruction and pedagogical questions. In the storybooks, where pedagogical questions were inserted, direct instructions were removed in order to control for book length and amount of text. In this way, the *Pedagogical Questions* condition served as a strong comparison to the *Direct Instruction* condition. Despite the fact that children in the *Pedagogical Questions* condition were given less relevant information about psychosomatic events than children in the *Direct Instruction* condition, they still demonstrated greater learning and transfer of the causal mechanism, and improved memory for other story content.

In this way, the current study maps onto the broader literature on playful learning, which demonstrates that forms of guided play can be equally or more effective than direct instruction in bolstering learning outcomes and engagement in young children^12,13^. Guided play has also been referred to as “enhanced learning”, “inquiry-based learning”, “guided discovery learning”, and even more broadly, “child-centered learning” among many other terms^12,14–17^. The multitude of terms used to describe guided play is indicative of the fact that this construct is also inconsistently defined and measured across studies. Thus, the current study aimed to isolate the effect of one specific element (PQs) of guided play in order to test its unique contribution to children’s learning. The current findings suggest that pedagogical questions are a crucial element when promoting learning through guided play, however, more studies are needed in order to further refine the cognitive mechanisms by which pedagogical questions specifically, and guided play more broadly, may improve learning.

This work also provides a new perspective on the role of explanation in learning. It has been suggested that explanations promote metacognitive awareness by highlighting conflicts between explanations and beliefs or evidence^18–20^, thus making learning more active. Furthermore, researchers have suggested that explanations also help learners focus their attention on relevant information, evidence, or features by engaging them in the process of learning^21^, which may help them develop a more sophisticated understanding of the acquisition of knowledge^22^. Interestingly, explanations are typically prompted by posing a pedagogical question to the learner. Although we do not directly contrast pedagogical questions with other types of questions here, our work raises the possibility that the pedagogical nature of an explanatory prompt may be a substantial source of these active learning benefits. Of course, it is likely that additional benefits may come about by explaining as well.

The literature on active learning suggests that active engagement improves memory^2^ and academic outcomes compared to more traditional, passive forms of learning like direct instruction^3,4^. It is important to note that the current study is unable to address the possibility that improved memory is the mechanism underlying the effectiveness of PQs for learning. However, the present results do provide further support for the hypothesis that active engagement improves memory. However, what encourages “active engagement” has been less well defined in the field. Typically, this involves allowing the learners choice of which information to see next^2,23^. We suggest, in addition, that the kind of active engagement that leads to improved learning can also be fostered through question-asking.

Previous research has investigated the use of questions in learning. For example, our work connects to a broader literature on the role of question-asking in academic outcomes^24,25^ and learning more broadly^26,27^. Asking children questions supports language learning^28,29^, and leads to general content knowledge^30^. Child-directed questions are crucial mechanisms by which children can explore and learn^31^. However, not all questions are created equal. For example, the current study did not include an information seeking question condition because previous work has shown that children do not treat questions asked by a naive other as “pedagogical”, and thus do not demonstrate the increased exploration as with pedagogical questions^7,8^. Here we build on this past work by demonstrating that pedagogical questions contribute to children’s learning above a direct instruction-only approach. In other words, children’s learning is bolstered when pedagogical questions are introduced even at the expense of direct instruction. It is important to note that the current study does not distinguish between the benefits of pedagogical questions from the benefits of other types of questions, such as information seeking questions. It may be the case that question-asking in general—not just pedagogical question-asking—promotes young children’s learning. Future work should contrast pedagogical questions with other types of questions to better understand the mechanisms by which question-asking facilitates learning.

Our results show that pedagogical questions are a relatively simple manipulation to influence learning, the retention of learned material, and the generalization of learned concepts by children. It is commonplace that educational materials present facts as declarative, didactic instruction. Pedagogical questions are a straightforward adaptation making information more engaging and increasing learning. Pedagogical questions thus offer a promising avenue for simple educational interventions.

## Methods

All experimental protocols were approved by Arts and Sciences IRB New Brunswick in Office of Research Regulatory Affairs, Rutgers University (Learning, Perception, and Belief Revision in Infants, Children, and Adults, Protocol ID: 16-625Mc), and all methods were carried out in accordance with relevant guidelines and regulations. Additionally, all procedures and analyses were preregistered at AsPredicted (Training psychosomatic understanding with pedagogical questions—#10599: http://aspredicted.org/blind.php?x=p28qt4). Participants were recruited from preschools that serve families from diverse racial and socioeconomic backgrounds in a Northeastern metropolitan area. Parental consent forms were sent home with all children attending participating preschools, and only children who received informed consent from a parent and/or legal guardian to participate and who provided verbal assent to an experimenter were allowed to participate in the study.

Participants were 72 preschoolers ranging in age from 42- to 54-months-old (*M*_age_ = 49 months; 50% female). Four participants who did not speak English were excluded and replaced for a final sample of *N* = 72. Participants participated one-on-one with an experimenter in quiet spaces in their preschools designated by preschool directors. All participants completed two training sessions and a posttest during three 15-min visits within a 2-week period. During the first visit, immediately following the administration of the pretest screener storybook, the experimenter read three training books to the children. During the second visit, the experimenter read the training books with children a second time. During the third visit, children completed the posttest, which contained measures of children’s psychosomatic understanding, memory for storybook details, and storybook preference.

Participants read a pretest screener storybook with an experimenter to ensure that they did not already endorse psychosomatic events. In this book, the protagonist (a bunny) repeatedly displayed a biological effect (having a tummy ache) after both biological events (eating and drinking certain things) and a psychological event (e.g., feeling worried; adapted from Ref.^10^; Fig. 1). The book was designed in such a way that statistically, the psychological event is the more likely cause of the biological effect. At the end of the book, the bunny gets one final tummy ache, and children are asked, “Can you tell me why you think bunny has a tummy ache? Is it because bunny ate the cornbread, bunny drank the papaya juice, or bunny feels worried?” Children who attributed the bunny’s tummy ache to a psychological cause (i.e., feeling worried about show-and-tell) were considered to have “passed” the screening, and were subsequently dropped from the study and replaced. Because we were interested in children’s ability to understand psychosomatic events, only children who attributed the bunny’s tummy ache to a physical cause (i.e., eating the cornbread) participated in the following intervention.

Children were randomly assigned to one of three conditions (*n* = 24 per condition): (1) *Direct Instruction* (DI), (2) *Pedagogical Questions* (PQ), and *Control* (CT). Each condition corresponded to one set of three training books. The *Direct Instruction* books taught children possible causal mechanisms of psychosomatic events [adapted from the *Mechanism training* condition of Ref.^11^), and are written only in factual statement form. The *Pedagogical Questions* books were identical to the *Direct Instruction* books, except that some of the statements were replaced with questions about the same information. For example, “When you feel happy, you might also smile.” in the *Direct Instruction* condition was changed to “When you feel happy, might you smile?” in the *Pedagogical Questions* condition. The *Control* storybooks followed the same protagonists and the same storylines, except characters’ psychosomatic experiences were not discussed. Book length and the total number of words in each book were held constant across all conditions.

During the third visit, children completed the posttest, which contained measures of children’s psychosomatic understanding, memory for storybook details, and storybook preference. Children’s psychosomatic understanding was measured using (1) a modified storybook, (2) a free explanation storybook, and (3) judgments of possibility of psychosomatic events. Memory for storybook details was assessed for causally relevant and irrelevant content. Book preferences were assessed for new books.

### Measures

#### Psychosomatic understanding

Measures of psychosomatic understanding included a modified storybook, free explanation book, and psychosomatic possibility judgments.

##### Modified storybook

During posttest, the experimenter first read a modified storybook that was structurally identical to the pretest storybook screener, but with a different protagonist (i.e. a beaver nervous about going to the dentist) and a different set of causes and effects. At the end of the book, the experimenter asked children what they thought the cause for beaver’s belly ache was, and children were provided with a 3-item forced-choice response (one psychological cause and two biological causes). The dependent variable was children’s choice (1 for choosing the psychological cause, 0 for choosing the biological causes).

##### Free explanation storybook

In its entirety, this book read: “This is Puppy. Puppy is nervous because it’s his first day of school. Oh, oh! Puppy’s stomach hurts!” Children were asked: “Why does Puppy’s stomach hurt?” In this way, children were asked to explain why they thought the puppy’s stomach hurt. If any of the training conditions support children’s ability to learn psychosomatic causality, then we may also see transfer of this ability to this novel, free explanation task. The dependent variable was whether children’s explanations to this prompt were psychosomatic in nature (i.e. “Because Puppy is nervous about school”).

##### Psychosomatic possibility

In the third task (adapted from Refs.^9,10^) the experimenter showed children pictures that describe someone who experiences certain psychological events such as feeling upset or embarrassed (e.g. “This is Jordan. Jordan is in his bedroom. Jordan is upset because he has to stay with a babysitter. Jordan feels very upset about the babysitter. Jordan has heard from his friend that if he keeps being upset he will start to feel sick.”). Then children were given a prompt, relating to whether it was possible for these experiences to lead to certain biological effects (e.g. “Do you think that could happen? Can Jordan feel sick from being upset?”). The dependent variable was children’s possible/impossible judgment for four such stories (1 for possible, 0 for impossible, score range 0–4). Based on past work, brief direct instruction training was insufficient to affect children’s overall impossibility judgments, but we included this far generalization task here to assess whether pedagogical questions may boost performance on this far generalization task.

##### Composite score

Composite scores for children’s performance on the modified storybook, free explanation storybook, and the possibility judgments were created using standard scores. For ease of interpretability, the *z*-scores were then transformed so that the *z*-score of the control group was equal to zero (see Table 1 and Fig. 2).

#### Memory for storybook details

The next task was a memory test that asked about details from the training books. For each training book, there were two questions: one pertaining to causally-*relevant* details of the story (e.g., Do you remember what happened to Gail in this story?” and if children needed a prompt, “Did Gail win a soccer game or lose a basketball game?”), and one pertaining to causally-*irrelevant* details of the story (e.g., “Do you remember his/her name?”, and if the child needed a prompt, “Was her name Gail or was her name Kayla?”). Children’s responses were coded for accuracy. For both types of questions, children received 2 points for answering correctly without a prompt, 1 point for answering correctly with a prompt, and 0 points for answering incorrectly even after receiving a prompt. The total possible score was 12 (6 questions; 2 points per question) (Supplementary Information).

#### Children’s storybook preferences

The last task measured children’s preference among new *Direct Instruction*, *Pedagogical Questions*, and *Control* books. We included this measure to rule out the possibility that any effect of PQs on learning, generalization, and memory could not be interpreted as simple child *preference* for books containing PQs. In this task, we provided children with a forced response task, in which we read the titles of each of three new books with novel titles to the children, and then asked children which book they would prefer to read. Specifically, the experimenter laid all three books out on the table so that the child could clearly see all of the books entitled *What Makes Sam Stomp His Feet?* (PQ), *Feeling Angry Makes Sam Stomp His Feet* (DI), and *Mom Makes Sam Brush His Teeth* (CT). The PQ book contained pedagogical questions about how feeling angry can make Sam want to stomp his feet, in contrast to the DI book, which covered the same topic, but in the form of statements instead of questions. The CT book story was about Sam, a boy with dirty teeth, whose mother made him brush them. Then the experimenter said, “We only have time to read one book, so I want you to choose the book that you would like to read”, and the child and experimenter would read that book. Next, the experimenter would say, “Oh! It looks like we have more time than I thought. Which book would you like to read next?” The child and the experimenter would read the second book the child selected, and then finally the third book. Book length and the total number of words in each book were held constant across all books. Books were ranked first to third in order of preference for each child. The dependent variable was the book children choose to read first. We computed and compared the percentage of children who preferred each book.

## Supplementary information

Supplementary Information.

## Data Availability

The data presented in this paper is available on the Open Science Framework (under embargo until publication).
