# Supplementary material for: Pedagogical questions promote causal learning in preschoolers
Source: Sci Rep. 2020 Nov 26;10:20700. doi: 10.1038/s41598-020-77883-5 (PMC7691986; doi:10.1038/s41598-020-77883-5)
Supplement: Supplementary file 1 — Supplementary Information. [file 41598_2020_77883_MOESM1_ESM.docx]

**Supplemental Material**

**Pedagogical Questions Promote Causal Learning in Preschoolers**

Emily N. Daubert, Yue Yu, Milagros Grados, Patrick Shafto, Elizabeth Bonawitz

**Supplementary Analyses**

In addition to the Analyses of Variance (ANOVAs) presented in the manuscript, post-hoc parallel analyses using Generalized Linear Mixed Models (GLMMS) and LMMs are presented here. Analyses were conducted to account for any random effects present in the model, especially those which may have arisen from the repeated nature of the outcome variables. For all following analyses, we used children’s responses in each trial as the dependent variable. Children’s psychosomatic understanding for each storybook was a binary variable and was fitted using logistic regression models. Their answers to memory questions ranged from 0 to 2 for each question, and therefore were fitted using linear regression models. We included condition as an independent variable with fixed effects, and participant as an independent variable with random effects. Condition was dummy coded; the Direct Instruction condition was used as the referent group in order to test the group differences of most interest (PQ vs. DI; DI vs. CT). For each dependent variable we first ran a null model which only included the random variable, before running a full model which included both random and fixed effect variables. All models were fitted using maximum likelihood (ML) method and not restricted maximum likelihood (REML) method to facilitate comparisons between models with different numbers of fixed effects.

***Psychosomatic Understanding***

First, GLMMs were conducted to test for group differences on children’s responses in the modified storybook and the free explanation story book. Compared to the null model, the full model showed a significantly better fit to the data, *χ*^2^ (2) = 14.6, *p* < .001. This confirmed the ANOVA results that children’s psychosomatic understanding was influenced by the condition they were assigned to. Specifically, the odds of children understanding psychosomatic causes was 2.65 times as high for children in Pedagogical Question condition as for children in Direct Instruction condition, and 5.66 times as high for children in Direct Instruction condition as for children in Control condition. Though consistent with the parallel ANOVA presented for the psychosomatic understanding composite in the manuscript, this result did not yield a significant group difference between children in the Pedagogical Question and children in the Direct Instruction group.  It is possible this is due to the fact that the ANOVA was conducted using planned contrasts, whereas this is not possible using GLMMs. Additionally, our sample size was estimated using the predictions from these planned contrasts, and so a GLMM is underpowered.

***Memory for Storybook Details***

**Overall Memory.** Second, LMMs were conducted to test for group differences on children’s memory for storybook details (relevant and irrelevant combined). Compared to the null model, the full model showed a significantly better fit to the data, *χ*^2^ (2) = 6.4, *p* = .04. This confirmed the ANOVA results that children’s memory was influenced by the condition they were assigned to. Children in the Pedagogical Question condition remembered significantly better than children in the Direct Instruction condition, *β* = 0.28, 95% CI = [0.06, 0.49]. There was no significant difference between children in the Direct Instruction and Control conditions, *β* = 0.08, 95% CI = [-0.13, 0.30]. These LMM results mirror exactly the pattern of results yielded by the parallel ANOVA presented in the manuscript.

***Relevant Versus Irrelevant Storybook Details***

Focusing only on relevant story details, the null and full LMMs revealed that the effect of condition was marginally significant, *χ*^2^ (2) = 5.5, *p* = .06. Children in the Pedagogical Question condition remembered significantly more than children in the Direct Instruction condition, *β* = 0.35, 95% CI = [0.05, 0.65]. There was no significant difference between children in the Direct Instruction and Control conditions, *β* = 0.07, 95% CI = [-0.23, 0.37]. In contrast, there were no significant differences between the null and full LMM models on irrelevant storybook details, *χ*^2^ = 3.5, *p* > .10. These results mirror the parallel ANOVAs in the manuscript.

***Exploratory Analysis***

Finally, an exploratory GLMM predicting psychosomatic understanding, after controlling for children’s memory for relevant storybook details was conducted. This analysis could provide support for the possibility that pedagogical questions improve learning by improving memory for relevant content. We compared three models of children’s responses in the modified storybook and the free explanation story book: The null model included only participant (random effect) and children’s age (fixed effect); The reduced model included participant, age, and the total score for children’s memory for relevant details (fixed effect); The full model included participant, age, relevant memory, as well as condition (fixed effect). See Table S1 for model summaries. Results revealed no significant improvement of fit for the reduced model compared to the null model, *χ*^2^ (1) = 0.01, *p* > .20, which suggests that children’s memory of relevant information did not predict their psychosomatic understanding. On the other hand, the full model fitted the data significantly better than the reduced model, *χ*^2^ (2) = 15.0, *p* < .001, which suggests that condition has a significant impact on children’s psychosomatic understanding, after controlling for memory. Specifically, the odds of children understanding psychosomatic causes was 3.01 times as high for children in Pedagogical Question condition as for children in Direct Instruction condition, and was 5.43 times as high for children in Direct Instruction condition as for children in Control condition. Overall, these analyses are consistent with the possibility that an independent variable explained condition differences, separate from memory (which may correlate with performance due to this independent factor, or due to some other factor).

| **Model** | **Number of parameters** | **AIC** | **BIC** | **Log likelihood** | **Deviance** |
| --- | --- | --- | --- | --- | --- |
| Null | 3 | 145.07 | 153.98 | -69.537 | 139.07 |
| Reduced | 4 | 147.07 | 158.95 | -69.534 | 139.07 |
| Full | 6 | 136.06 | 153.88 | -62.029 | 124.06 |

**Table S1. Model summaries for the null, reduced, and full models.** Model summaries for the GLMMs predicting psychosomatic understanding, after controlling for children’s memory. See Supplemental Material for a full discussion of these analyses.
